# Microglia in post-mortem brain tissue of patients with bipolar disorder are not immune activated

**DOI:** 10.1038/s41398-019-0490-x

**Published:** 2019-05-24

**Authors:** Marjolein A. M. Sneeboer, Gijsje J. L. J. Snijders, Woutje M. Berdowski, Alba Fernández-Andreu, Psychiatric Donor Program of the Netherlands Brain Bank (NBB-Psy), Hans C. van Mierlo, Amber Berdenis van Berlekom, Manja Litjens, René S. Kahn, Elly M. Hol, Lot D. de Witte

**Affiliations:** 1 Department of Psychiatry, Brain Center Rudolf Magnus, University Medical Center Utrecht, Utrecht University (BCRM-UMCU-UU), 3584 CG Utrecht, The Netherlands; 2 Department of Translational Neuroscience, Brain Center Rudolf Magnus, University Medical Center Utrecht, Utrecht University (BCRM-UMCU-UU), 3584 CG Utrecht, The Netherlands; 3 Psychiatric Donor Program of the Netherlands Brain Bank (NBB-Psy), Meibergdreef 47, 1105 BA Amsterdam, the Netherlands; 4 Department of Psychiatry, Icahn School of Medicine at Mount Sinai, New York, NY USA; 5 Mental Illness Research, Education and Clinical Center (MIRECC), James J Peters VA Medical Center, Bronx, NY USA; 6 Neuroimmunology, Netherlands Institute for Neuroscience, an Institute of The Royal Academy of Arts and Sciences, 1105 BA Amsterdam, The Netherlands

**Keywords:** Molecular neuroscience, Bipolar disorder

## Abstract

Genetic, epidemiological, and biomarker studies suggest that the immune system is involved in the pathogenesis of bipolar disorder (BD). It has therefore been hypothesized that immune activation of microglia, the resident immune cells of the brain, is associated with the disease. Only a few studies have addressed the involvement of microglia in BD so far and a more detailed immune profiling of microglial activation is lacking. Here, we applied a multi-level approach to determine the activation state of microglia in BD post-mortem brain tissue. We did not find differences in microglial density, and mRNA expression of microglial markers in the medial frontal gyrus (MFG) of patients with BD. Furthermore, we performed in-depth characterization of human primary microglia isolated from fresh brain tissue of the MFG, superior temporal gyrus (STG), and thalamus (THA). Similarly, these ex vivo isolated microglia did not show elevated expression of inflammatory markers. Finally, challenging the isolated microglia with LPS did not result in an increased immune response in patients with BD compared to controls. In conclusion, our study shows that microglia in post-mortem brain tissue of patients with BD are not immune activated.

## Introduction

The aetiology of bipolar disorder (BD) is still largely unknown. Several lines of evidence suggest that the immune system is involved in the pathogenesis of BD. Epidemiological studies on large birth cohorts have shown an association between BD and autoimmune diseases, atopic disorders, and severe childhood infections^1–3^. Additionally, genetic studies found that several genes associated with BD cluster in immune pathways^4^. Furthermore, many research groups have reported alterations in peripheral immune markers in BD, with increased levels of C-reactive protein (CRP) and several cytokines, such as IL1β, IL6, and TNFα, in blood and cerebrospinal fluid of patients with BD^5–7^. Besides, a decreased percentage of T-regulatory cells^8–10^ and an altered gene expression profile of circulating monocytes in the blood have been described^8,11^.

It is not yet understood how these immune associations are involved in the pathogenesis of BD, but activation of microglia, the immune cells that reside in the brain parenchyma, has been hypothesized to play a central role^12–15^. Microglia are part of the innate immune system and have an important function in initiating and controlling neuroinflammation in the CNS via the secretion of pro- and anti-inflammatory cytokines. In addition, these cells are involved in neurodevelopment and neuronal functioning in adulthood by promoting synaptogenesis and synaptic pruning^16,17^. Besides, microglia are involved in the regulation and production of serotonin of which an imbalance has been implicated in BD^13^. In homeostatic conditions, microglia have a ramified morphology with highly motile protrusions that scan the environment for possible danger signals, such as pathogens or cell debris. Upon detection, microglia become immune activated. They migrate to the site of injury, and change their expression profile and shape to a more rounded or so-called ‘amoeboid’ morphology. This process is necessary to adequately respond to the insult via the secretion of cytokines and chemokines, antigen presentation of the microglia, and apoptosis of the injured cell^18–20^. Although microglial immune activation is crucial to minimize the effects of the injury, prolonged activation will lead to the release of reactive oxygen species, which are toxic to neurons and other neighbouring cells^21^. In BD it has been hypothesized that microglia are immune activated, possibly induced by circulating peripheral immune factors, and result in CNS damage and abnormalities^22,23^.

However, evidence for immune activation of microglia in BD is still lacking. Five post-mortem studies investigated the density of microglia or the expression of microglia markers in BD. The samples sizes ranged from 9 to 20 subjects, different microglial markers were used, and multiple brain regions were studied. None of the studies found an increased microglial density or elevated expression of microglial markers^24–27^. One study even found decreased mRNA expression of the microglial markers *ITGAM* (CD11b) and *CD68* in the anterior cingulate cortex and prefrontal cortex^28^. Moreover, in a positron emission tomography study with the [(11)C]-(R)-PK11195 tracer, there was no sign for microglia activation in most regions of the brain in BD, except for the hippocampus^29^. A drawback of these studies was that most used one methodological approach (i.e., number of microglia, microglial mRNA expression or protein level), non-automated analysis and focussed on immunostainings that only give limited information about the diversity of microglial phenotype and function. To conclude, an in-depth microglial immune profile examining more specific signs of microglial activation is still missing in BD.

Therefore, the aim of this study was to determine whether microglia in post-mortem brain tissue of patients with BD are immune activated. We applied a multi-level approach and characterized microglial density, and inflammatory functions in the medial frontal gyrus (MFG) of patients with BD. To further profile the microglia and examine their phenotype and functions, we isolated human primary microglia from fresh post-mortem brain tissue of the MFG, superior temporal gyrus and thalamus. This allows us to characterize microglia outside the brain and assess their functions more extensively^30–33^. This exclusive approach has been used to profile microglia in neurologic disorders, such as multiple sclerosis and Alzheimer’s disease^34–36^, but has not yet been applied to psychiatric disorders. To our knowledge, the present study is the first investigating microglial immune activation in ex vivo isolated human microglia in combination with immunohistochemistry and mRNA profiling.

## Materials and methods

### Donors

Paraffin (control *N* = 12; BD *N* = 16), snap frozen (control *N* = 16; BD *N* = 15), and fresh post-mortem brain tissue (control *N* = 19; BD *N* = 12) of patients with BD and controls was obtained from the Netherlands Brain Bank (www.brainbank.nl). The permission to collect human brain material was obtained from the Ethical Committee of the VU University medical center (VUmc, Amsterdam, The Netherlands). An overview of the clinical characteristics and donors used per experiment is summarized in Table 1 and Supplementary Table 1. We selected the medial frontal gyrus (MFG) as the main region of interest (ROI). For fresh post-mortem brain tissue, we also selected the superior temporal gyrus (STG) and thalamus (THA). All three regions have been associated with BD^20,37,38^. Permission for brain autopsy and the usage of brain tissue and accompanied clinical information for research purposes was obtained per donor ante-mortem. Due to the insufficient quality of the tissue or limited number of viable microglia after isolation, we could not include every donor for all downstream analyses. Control donors are defined as donors without a history of major psychiatric diseases, confirmed by retrospective medical chart review. There was no significant difference between controls and patients with BD in age, post-mortem delay (PMD), and pH (Table 1). Only in frozen tissue, sex was significantly different in both grey (*p* = 0.004) and white matter (*p* = 0.008) between patients with BD and controls.

**Table 1:** Summary of clinical information and post-mortem variables of the study

		Control (*N* = 12)	BD (*N* = 16)
Immunohistochemistry (paraffin tissue)	Age (years)	75.6 ± 11.8	72.0 ± 10.4
	Sex (M:F)	7:5	12:4
	PMD (minutes)	566 ± 376	429 ± 176
	pH	6.53 ± 0.18	6.45 ± 0.18
		Control (*N* = 16)	BD (*N* = 15)
mRNA expression (frozen tissue)	Age (years)	75.06 ± 13.02	74.73 ± 8.04
	Sex (M:F)	5:11*	12:3*
	PMD (minutes)	429 ± 189	430 ± 183
	pH	6.52 ± 0.29	6.42 ± 0.23
		Control (N = 16)	BD (N = 12)
mRNA expression (isolated microglia)	Age (years)	79.69 ± 11.97	74.91 ± 17.10
	Sex (M:F)	5:11	5:7
	PMD (minutes)	447 ± 128	471 ± 120
	pH	6.75 ± 0.32	6.66 ± 0.45
		Control (*N* = 17)	BD (*N* = 9)
Protein expression (isolated microglia)	Age (years)	83.12 ± 10.39	73.22 ± 17.26
	Sex (M:F)	6:11	3:6
	PMD (minutes)	447 ± 122	472 ± 128
	pH	6.35 ± 0.29	6.66 ± 0.45
		Control (*N* = 19)	BD (*N* = 9)
LPS response (isolated microglia)	Age (years)	79.44 ± 11.8	73.57 ± 19.69
	Sex (M:F)	6:13	3:6
	PMD (minutes)	430 ± 124	447 ± 117
	pH	6.72 ± 0.30	6.83 ± 0.48

Summary of the clinical information (age and sex) and post-mortem variables (post-mortem delay (PMD) and pH) of donors used in the study. Information is separated per experimental category. Numbers represent mean ± standard deviation

*M* males, *F* females, *BD* bipolar disorder

*Significantly different between patients with BD and controls in grey (*p* = 0.004) and white matter (*p* = 0.008)

### Immunostaining and image analysis

Paraffin-embedded tissue of the MFG of patients with BD and controls was sectioned at 7 μM. The sections were deparaffinised using a standard xylene and alcohol series, followed by blocking of endogenous peroxidase with PBS, 1% H_2_O_2_ (Merck, Germany). For antigen retrieval, sections were heated in 0.01 mM citrate buffer (Merck, Darmstadt, Germany), 0.05% Tween-20 (Merck, Darmstadt, Germany), pH = 6.0 for 15 min. Subsequently, aspecific binding was blocked in PBS with 1% normal horse serum (NHS, Thermo Fisher Scientific, Massachusetts, USA), 0.1% bovine serum albumin (BSA, Merck, Darmstadt, Germany), 0.2% Triton X (Merck, Darmstadt, Germany). Sections were incubated with a rabbit polyclonal anti-Iba1 antibody (Wako Pure Chemical Industries, Ltd., 1:1000) at 4 °C. Next day, secondary goat-anti-rabbit biotin (Jackson ImmunoResearch Laboratories, Inc., 1:400) was added, followed by avidin-biotin-peroxidase (AB) complex (Vector Laboratories, USA). To visualize the microglia, the sections were incubated with a 3,3′ diaminobenzidine (DAB) substrate (DAKO, USA). Finally, tissue sections were dehydrated using an alcohol and xylene series and embedded in Entellan (Merck, Darmstadt, Germany). Per tissue section, six pictures of 89,44 um × 119,37 um were taken randomly and blinded for diagnosis from grey and white matter. Two investigators (MS and GS) separately counted the number of microglia in each picture manually and averaged the total number of counted microglia per donor. Microscopic images were also analysed automatically using open source software ImageJ. Microglial cell density was calculated with a particle analysis macro-script, by dividing the cell numbers by the measured area (microglia/mm^2^) (for further details see Supplementary Methods). There was a high level of correlation between manual cell counts and automated cell counts (R_s_ = 0.780, *p* < 0.05) and Cronbachs alpha was greater than >0.80 (CB alpha 0.879). Immunofluorescent staining was performed additionally to determine the number of microglia relative to total cell number. The first part of the staining procedure was similar as for DAB-immunostained microglia, with the usage of donkey anti-rabbit Alexa 488 (1:700) and Hoechst (1:1000) as secondary antibody. An automated macro script was used to quantify the number of microglia (Iba1+Hoechst+) or total cell number (Hoechst+) (See Supplementary Fig. 1a and Supplementary Methods).

### Human primary microglia isolation

Human primary microglia (pMG) were isolated from fresh post-mortem brain tissue of the different ROIs based on an earlier established protocol^30^. Brain tissue was first mechanically dissociated through a metal sieve in a glucose- potassium-sodium buffer (GKN-BSA; 8.0 g/L NaCl, 0.4 g/L KCl, 1.77 g/L Na_2_HPO_4_.2H_2_O, 0.69 g/L NaH_2_PO_4_.H_2_O, 2.0 g/L D-(1)-glucose, 0.3% bovine serum albumin (BSA, Merck, Darmstadt, Germany); pH 7.4) and supplemented with collagenase Type I (3700 units/mL; Worthington, USA) and DNase I (200 µg/mL; Roche, Switzerland) at 37 °C for 30 min (THA) or 60 min (MFG, STG) while shaking. The suspension was put over a 100 µM cell strainer and washed with GKN-BSA buffer in the centrifuge (1800 rpm, slow brake, 4 °C, 10 min) before the pellet was resuspended in 20 mL GKN-BSA buffer. 10 mL of Percoll (Merck, Darmstadt, Germany) was added dropwise and the tissue homogenate was centrifuged at 4000 rpm (fast acceleration, slow brake at 4 °C, 30 min). The middle layer was collected and washed with GKN-BSA buffer, followed by resuspension and centrifuging in a magnetic-activated cell sorting (MACS) buffer (PBS, 1% heat-inactivated fetal cow serum (FCS), 2 mM EDTA; 1500 rpm, 10 °C, 10 min). Microglia were positively selected with CD11b conjugated magnetic microbeads (Miltenyi Biotec, Germany) according to the manufacturer’s protocol. This method relies on the membrane expression of CD11b, which is also present on perivascular and infiltrated macrophages in the central nervous system (CNS)^30^. Mass cytometry analysis has shown that the percentage of perivascular macrophages (CD206high) was low (<2%) in microglia isolated from control donors and individuals with psychiatric disorders, suggesting that majority of cells that we analyze are indeed microglia^39^. Microglia were lysed in TRIzol reagent (Invitrogen, USA), stained for flow cytometry or cultured in a poly-l-lysine (PLL; Merck, Germany) coated 96-wells flat bottom plate (Greiner Bio-One, Austria) at a density of 1.0 × 10^5^ cells in a total volume of 200 μL Rosswell-Park-Memorial-Institute medium (RPMI; Gibco Life Technologies, USA) supplemented with 10% FCS, 2 mM l-glutamine (Gibco Life Technologies, USA), 1% penicillin–streptomycin (Gibco Life Technologies, USA) and 100 ng/ml IL-34 (Miltenyi Biotech, Germany). After overnight incubation, pMG were stimulated with 100 ng/mL lipopolysaccharide (LPS) from Escherichia coli 0111:B4 (Merck, Germany) for 6 h. We selected 6 h LPS stimulations for its robust ‘inflammatory’ effect on microglia^30^. The concentrations and incubation times are based on dose-response curves^31^. The cells were harvested with TRIzol reagent and stored at −80 °C for further analysis of mRNA expression.

### Gene expression analysis

Grey and white matter were manually separated from a 50 µM section of frozen brain tissue at the cryostat and lysed with TRIzol reagent. RNA extraction, cDNA synthesis, and qPCR on these brain tissue samples and pMG were performed as described before^30^. Considering the low yield of RNA and the consistency when comparing single-well experiments with triplicates, we ran single-well experiments for each reaction. Primer sequences are listed in Supplementary Table 2. Absolute expression was calculated using the ΔΔCT method^40^. For the selection of reference genes we used the method described by Van den Sompele in 2002^41^. Expression Suite software 1.0.4 was used to select the most stable genes across ROIs, diagnosis, grey/white matter tissue and different donors for normalization. 18S ribosomal RNA (*18S*) and glyceraldehyde 3-phosphate dehydrogenase (*GAPDH*) were most stable for whole brain tissue and β-Actin (*ACTB*) and *GAPDH* for directly isolated pMG. LPS stimulated pMG were normalized to *GAPDH*. Samples with Cycle Threshold (CT) values higher than two times standard deviation above the average of each reference gene were excluded for further analysis. The average mean CT values of total brain tissue samples were between 25 and 31, of pMG between 23 and 30, and LPS stimulated pMG between 19 and 25. Median levels of relative expression between BD and controls were compared using non-parametric tests.

### Flow cytometry of human primary microglia

12–20 × 10^4^ human primary microglia were stained in a v-bottom 96-wells plate (Greiner Bio-One, Austria) directly after isolation. pMG were washed with 100 µL PBS with 0.5% BSA (PBA) and incubated in 25 µL PBA with specific monoclonal or isotype control antibodies (Supplementary Table 3). After staining, the cells were washed with PBA, fixated with 4% paraformaldehyde (Riedel-de Haën, Germany) in PBS at room temperature and stored in the fridge for flow cytometry analysis the next day. All microglia samples were processed on a FACS Canto (BD Bioscience) with calibrated settings and similar voltage for the fluorescent channels. The geomean fluorescence intensity (MFI) was determined by subtracting the MFI of the isotype control from the MFI of the positive antigen staining.

### Statistical analysis

Statistical analysis was done with GraphPad Prism software (version 7) and SPSS IBM 23. All variables were tested for homogeneity of variances and normality of distribution by means of the Levene and Kolmogorov–Smirnov tests, respectively. Differences between groups were analysed using chi-square (χ^2^) tests, independent *t*-tests or the Mann–Whitney *U* when appropriate. χ2 were used to test for sex difference and, independent *t*-test for age, PMD and pH differences. Non-parametric Mann–Whitney *U* test was performed to analyse differences in microglia density, gene expression, and protein levels between BD and controls, since data were non-normally distributed. Results were considered significant if the two-sided *p*-value was ≤0.05. Bonferroni correction for multiple testing was used for the qPCR and flow cytometry experiments, since more than one marker was analysed per region in the same experiment. For all outcome measures in this study, a spearman ranks correlation was performed to assess whether sex, age, PMD or pH were associated with the outcome. In case we found a significant association (Supplementary Table 4), an ANCOVA was applied to correct for possible confounding effects.

## Results

### Microglial density and morphology, and expression of microglial markers in total brain tissue

**Fig. 1:**
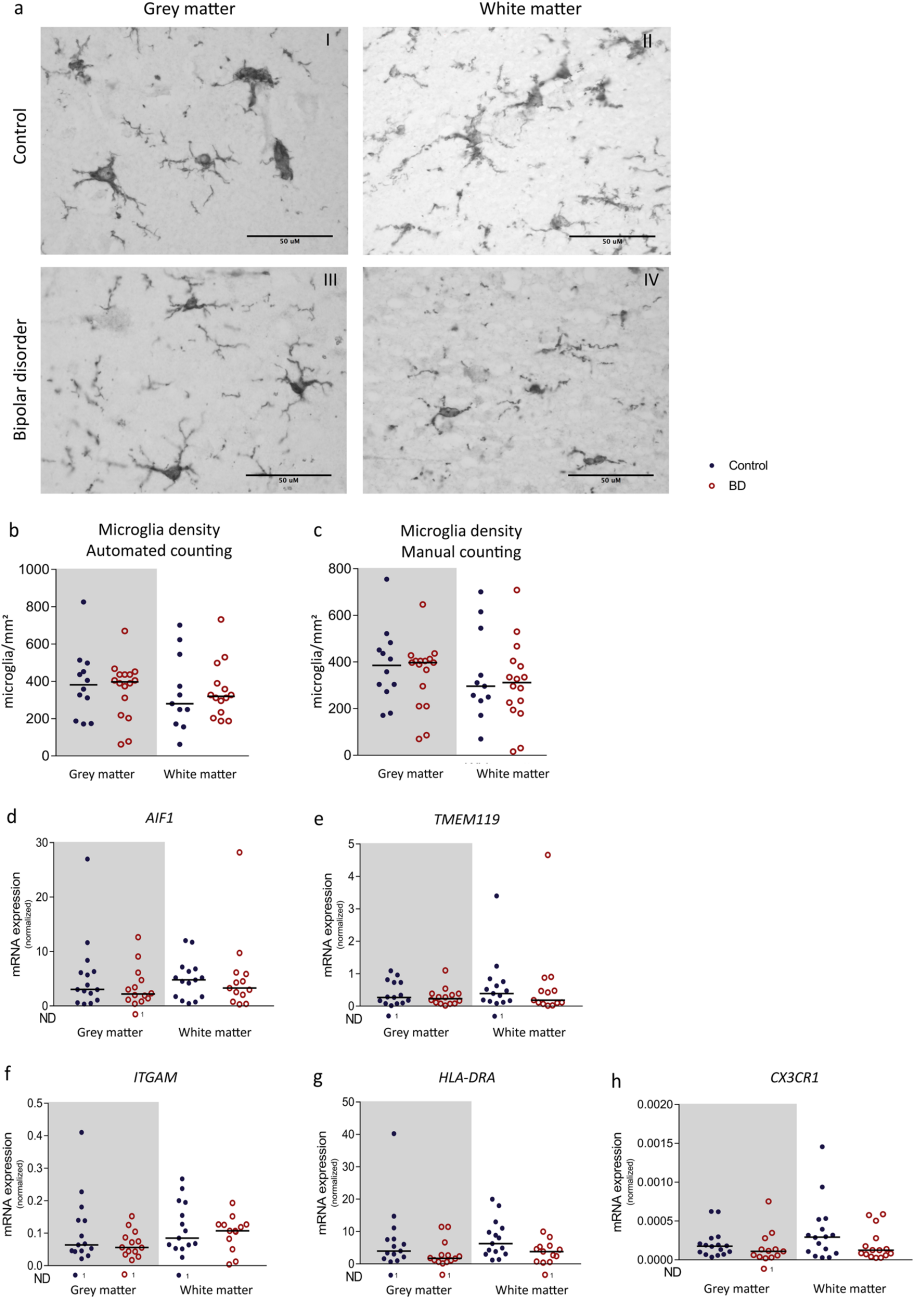
Microglial density, and expression of microglial markers in total brain tissue. **a–c** The medial frontal gyrus of patients with bipolar disorder (BD) and controls was stained for microglial marker ionized calcium-binding adaptor molecule (Iba1) to analyse microglial density and morphology. **a** Representative pictures are shown of microglia in grey (I, III) and white (II, IV) matter of controls (I–II) and patients with BD (III–IV). **b** The number of microglia (microglia/mm^2^) was manually quantified in grey and white matter in controls (*N* = 12, blue dots) and patients with BD (*N* = 12, red circles). **c** The number of microglia (microglia/mm2) was automatically quantified in grey and white matter in controls (*N* = 12, blue dots) and patients with BD (*N* = 16, red circles). **d**–**h** mRNA expression of microglial markers *AIF1* and *TMEM119*, myeloid marker *ITGAM* and *CX3CR1* and *HLA-DRA* in patients with BD (*N* = 15) and controls (*N* = 16) was determined using qPCR. Gene expression was normalized to 18S ribosomal RNA (18S) and glyceraldehyde 3-phosphate dehydrogenase (GAPDH) using the ΔΔCT method. Graphs show median expression levels and non-parametric testing with Bonferroni correction for multiple testing was performed. ND = number of non-detected samples

We analysed microglial density with immunohistochemistry using microglial marker ionized calcium-binding adaptor molecule 1 (Iba1). Figure 1a shows four representative pictures of DAB-Iba1^+^ microglia in the grey (I, III) and white matter (II, IV) in the MFG of a control donor (I–II) and patient with BD (III–IV). Manual and automated counting with ImageJ resulted in a similar microglial density between patients with BD and controls (Fig. 1b, c). Results were validated with immunofluorescent staining, showing no differences in microglial number relative to total cell number in BD compared to controls (Supplementary Fig. 1a, b). Furthermore, we assessed whether the expression of a panel of microglial-related or immune activated genes is changed in both grey and white matter of the MFG in BD. We defined three gene categories: (1) microglial-associated (*AIF1*, *P2Y12*, and *TMEM119*), (2) expressed on all myeloid cells (*CD68, CX3CR1* and *ITGAM*), and (3) (microglial)-immune activation (*HLA-DRA, IL1B* and *IL6)* (Table 2 and Fig. 1d–h). We did not find differences in expression between patients and controls for any of these genes.

**Table 2:** mRNA expression of microglial genes in the medial frontal gyrus

	Control		BD		Control			BD		
Gene	Median	IQR	Median	IQR	*p*-value	Median	IQR	Median	IQR	*p*-value
*AIF1*	3.05	6.35	2.19	4.75	0.68	4.78	6.81	3.28	5.88	0.43
*P2Y12*	0.86	3.08	0.74	1.64	0.50	1.22	2.95	1.48	1.78	0.85
*TMEM119*	0.27	0.73	0.23	0.38	0.78	0.39	0.75	0.19	0.48	0.43
*CD68*	1.31	2.89	0.58	1.44	0.30	2.88	3.69	1.25	1.69	0.10
*CX3CR1*	1.76E−4	1.83E−4	8.61E−5	1.44E−4	0.10	2.93E−4	4.00E−4	1.23E−4	2.20E−4	0.15
*ITGAM*	0.06	0.14	0.06	0.09	0.47	0.08	0.19	0.11	0.13	0.85
*IL1B*	0.05	0.34	0.06	0.20	0.71	0.18	0.38	0.22	0.31	1.00
*IL6*	0.04	0.16	0.05	0.17	0.45	0.05	0.12	0.15	0.33	0.52
*HLA-DRA*	3.97	7.71	1.74	2.73	0.20	6.25	11.03	3.81	5.28	0.06

mRNA expression was determined in grey (left) and white matter (right) of the medial frontal gyrus in patients with bipolar disorder (BD, *N* = 15) and controls (*N* = 16) by qPCR. Median, interquartile range (IQR; 25–75%) and *p*-value is shown for microglial-associated genes (*AIF1*, *P2RY12*, *TMEM119*), genes expressed on all myeloid cells (*CD68*, *CX3CR1, ITGAM*) and genes involved in (microglial) immune activation (*IL1B*, *IL6*, *HLA-DRA*)

### Phenotype and function of isolated microglia

**Fig. 2:**
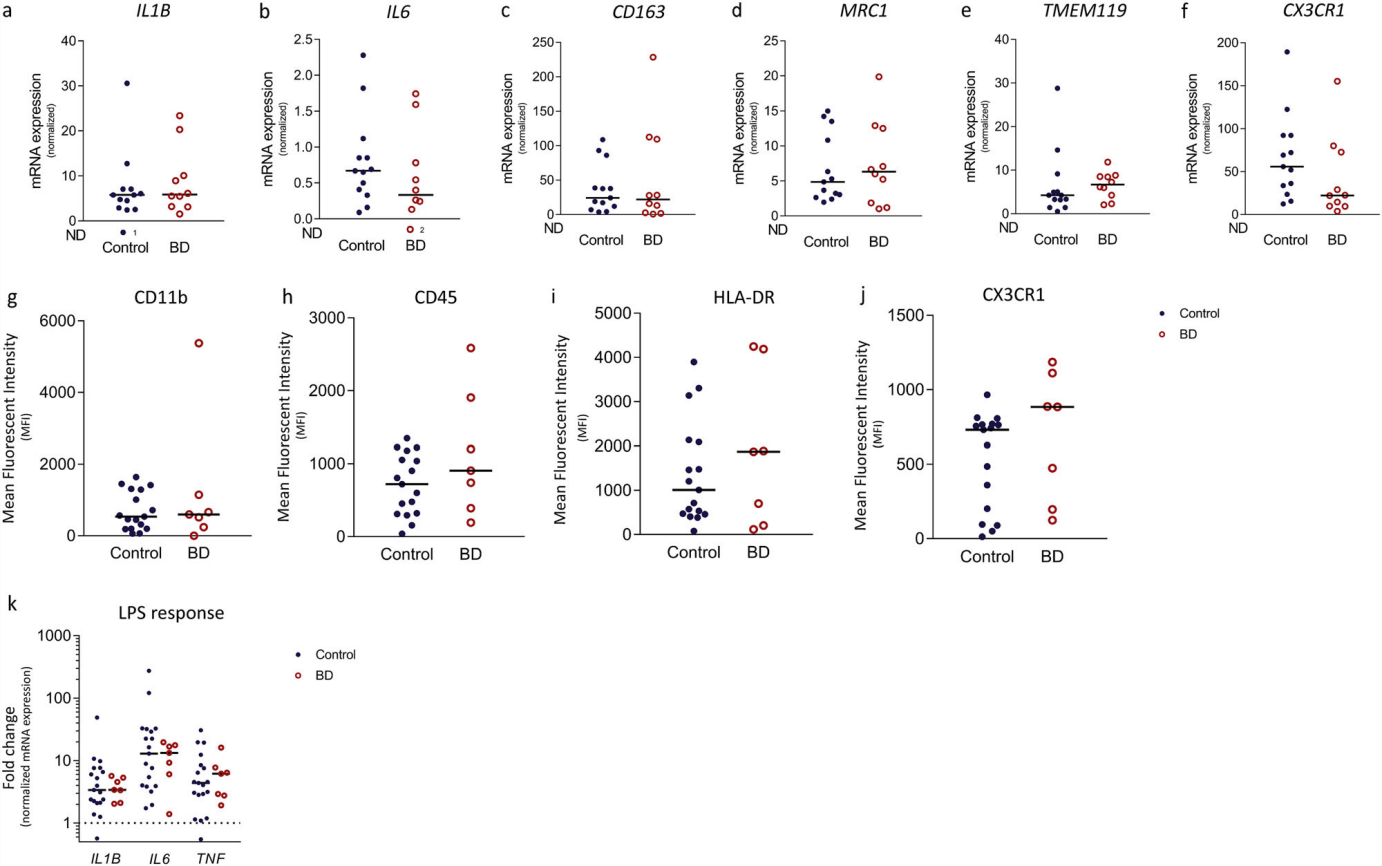
Phenotype and function of isolated microglia. Human microglia were isolated from the medial frontal gyrus of controls (*N* = 13, blue dots) and patients with bipolar disorder (BD, *N* = 10, red circles). **a–f** After isolation mRNA expression of pro-inflammatory genes (*IL1B* (**a**) and *IL6* (**b**)), anti-inflammatory genes (*CD163* (**c**) *and MRC1* (**d**)), the microglial specific gene *TMEM119* (**e**) and the myeloid marker *CX3CR1* (**f**) was determined in patients with BD and controls using qPCR. Gene expression was normalized to β-Actin (*ACTB*) and glyceraldehyde 3-phosphate dehydrogenase (*GAPDH*) using the ΔΔCT method. ND = number of non-detected samples. **g–j** Protein expression of isolated microglia from controls (*N* = 17) and patients with BD (*N* = 8) was characterized by flow cytometry. The mean fluorescent intensity (MFI) is determined for CD11b (**g**), CD45 (**h**) HLA-DR (**i**) CX3CR1 **j** Inflammatory response of isolated microglia from controls (*N* = 18 and patients with BD (*N* = 7) after 6 h of stimulation with lipopolysaccharide (LPS) was determined by measuring mRNA expression of *IL1B*, *IL6* and *TNF*. The fold change was calculated by dividing the mRNA expression of the LPS stimulated sample by mRNA expression of the non-stimulated sample of the same subject. The dotted line represents the baseline mRNA expression of non-stimulated cells. Graphs show median expression levels and non-parametric testing with Bonferroni correction for multiple testing was performed

Isolation of human primary microglia provided us with the opportunity to profile and characterize microglia as a pure cell population more extensively. We found no differences in the expression of genes involved in pro-inflammatory signalling (*IL1B* and *IL6*; Fig. 2a, b), genes related to anti-inflammatory functioning (*CD163* and *MRC1*; Fig. 2c, d), *TMEM119* (Fig. 2e), a microglial specific gene known for its homeostatic properties and *CX3CR1*, a gene related to microglial migration and neuron-glia interaction (Fig. 2f)^33,42^ in the MFG, STG, and THA (Supplementary Fig. 2). We analysed the expression of a panel of proteins by flow cytometry, including more general markers for microglia and myeloid cells (CD11b, CD11c, CD45, CD14, CD16, CD32, CD40, CD64, CX3CR1) and markers related to immune activation of the cells (CD163, CD172α, CD200R, CD206, HLA-DR, CD83, CD86) (Fig. 2g–j, Supplementary Table 5). Remarkably, the fluorescent mean intensity was quite variable between different donors. We found a significantly decreased expression of CD16 and CX3CR1 in the STG (Supplementary Table 5 and Supplementary Fig. 3d), however, this difference did not survive correction for multiple testing. We did not find differences in protein expression, including CD16 and CX3CR1, between controls and BD in the MGF and THA (Fig. 2j, Supplementary Fig. 3h and Supplementary Table 5).

To determine inflammatory responsiveness, we challenged the isolated human microglia with LPS and determined the mRNA expression levels of cytokines. Elevated expression of these markers in BD relative to controls would suggest increased (primed) immune activity of the cells. In contrast, less expression could indicate ‘silenced’ microglia in which sustained immune activation results in a blunted inflammatory response. We found that microglia from BD and controls both responded to LPS with increased expression of *IL1B*, *IL6*, and *TNF* (Fig. 2k). The effect was not significantly different between microglia of patients with BD and controls. The same was observed in microglia from the STG and THA (Supplementary Fig. 4).

## Discussion

The aim of this study was to elucidate whether microglia are immune activated in patients with BD, as is hypothesised by many studies^12–15,22,23^. We performed the most extensive, multi-level study to date in characterizing microglial inflammation in BD. We did not find any support for microglial immune activation, since we did not find differences in microglial density, and the expression of mRNA levels genes related to microglial function in fixed and frozen brain tissue. The absence of an immune activated phenotype of microglia in BD was confirmed by the lack of upregulation of inflammatory markers in ex vivo isolated human microglia on mRNA and protein level. Moreover, challenging human microglia of patients with BD with LPS did not result in an altered immune phenotype compared to control microglia. Altogether, the results of post-mortem studies, including ours, are consistent and do not support the hypothesis that microglia are immune activated in BD.

Our findings are in line with previous post-mortem studies that investigated microglial immune activation in BD by quantifying microglial cell numbers^24–27^. The results are also in agreement with recent transcriptomic studies on cortex lysates of BD patients and controls^43–48^. None of the activation markers that were examined in our present study were significantly different between BD and controls in any of these studies. For the two largest studies^47,48^ we extracted the results for these genes from their summary statistic files and depicted those in Supplementary Table 6. Interestingly, a downregulation of *CX3CR1, P2RY12, TMEM119, AIF1* and *ITGAM* was seen in these large studies. In addition, two smaller transcriptomic studies also found a downregulation of several microglial immune activation markers *(ITGAM, CX3CR1, AIF1, CD163* and *HLA-DR)* albeit not significant after correction for multiple testing^44,45^. In our data, we also found a trend towards downregulation of CX3CR1. A decreased expression of *CX3CR1* in grey (*p* = 0.10) and white (*p* = 0.15) matter MFG brain tissue was observed on a trend level (see Table 2 and Fig. 1h). Furthermore, we found a significantly decreased protein expression of CX3CR1 in isolated primary human microglia cells in the STG using flow cytometry (see Supplementary Table 5 and Supplementary Fig. 3d). Altogether, these studies indicate that although microglia are not immune activated, the phenotype and function of these cells might be changed. A downregulation of *CX3CR1*, *TMEM119* and *P2RY12* has also been described for stage 1 disease-associated microglia in neurodegenerative diseases^49^. Further studies are therefore warranted to analyse these changes in microglia phenotype in BD.

The hypothesis of immune activation as driving force in BD was mainly based on studies that have described immune alterations in the periphery^5–7^. This is in contrast to our results showing no microglial activation in the CNS, but could be explained in several ways. An important point is the difference in age between the patients in post-mortem studies and peripheral studies, with lower mean age in the latter. Therefore, we are only able to draw conclusions on microglial immune activation in the late phases of the disease. To further understand the relationship between peripheral immune alterations in BD and microglial activation an interesting follow-up study would be to analyse peripheral markers in serum and relate this to markers for microglial activation in the same post-mortem brain donors. At the same time, it is important to look at other explanations for the observed peripheral immune alterations in BD. Immune biomarkers in blood are very sensitive to changes in lifestyle, such as smoking, BMI, diet, stress and sleep problems^50,51^. We should, therefore, include the possibility that the peripheral changes actually reflect behavioural consequences and not the cause of the disease. On the other hand, this does not explain the increased prevalence of immune-related disorders in BD. Other immune-mediated mechanisms should, therefore, be investigated, such as autoimmunity and the role of non-inflammatory immune pathways in the brain that are for instance involved in glia-neuron communication^52^. It is becoming more evident that microglia have many more functions than controlling inflammation. Microglia are for example actively participating in modifying synapses in the brain^53,54^.

The multi-level approach used in this study contributed to a more detailed understanding of microglial activation in BD. Microglial heterogeneity was taken into account by making a distinction between microglia in grey and white matter brain tissue and by analysing isolated microglia from three brain regions. We applied different techniques to frozen, paraffin, and acutely isolated microglia to elucidate whether microglial immune activation is present. Additionally, we validated qualitative assessments of microglia density using scripted and automated analyses. A drawback of this study is the limited sample size per experiment (minimal six donors per condition). We performed a power analysis based on findings of earlier research^55^. We estimated that 34 subjects in each study arm would be sufficient to obtain a power of 80% at an α-level of 0.05. This number was not reached due to the rarity of the material. We included the maximum availability of brain tissue samples. Although we looked at microglia in three regions, it is also possible that microglial changes are restricted to specific areas or cortical layers of the brain. For the LPS stimulation study, we a priori selected three genes (*TNF, IL1B* and *IL6*) to determine the responsiveness of the microglia after 6 h. Quantification of genes other than these ‘inflammatory’ cytokines and long-term effects of LPS stimulations (>24 h) could be explored in future investigations. The availability of fresh post-mortem brain tissue is sparse, especially for psychiatric disorders. Post-mortem studies are subject to many confounders (e.g., chronicity of illness, use of psychotropic medication, somatic comorbidities such as neurologic, infection/autoimmune diseases), smoking, peri-mortem factors, and cause of death (euthanasia, suicide or somatic problems). Many of these factors have been shown to have a potential impact on microglia phenotype, such as mood stabilizers and antipsychotics^56,57^, neurological diagnoses^32,58^, aging^59^, environmental influences and lifestyles, including physical and mental activities, drugs, comorbidities and infections^60^, and suicide^61^.This makes primary changes intrinsic to the illness hard to distinguish from secondary, compensatory, and epiphenomenal effects. The absence of microglial activation might be concealed due to the effect of these confounding factors, however, confounder analyses for all these factors is not possible in our cohort due to small sample sizes. For some of our outcome measures, we found a significant correlation with one of the confounders age, sex, pH or PMD. However, correction for these factors resulted in similar findings. Controlling for medication was very difficult, due to the high variability of medication received by the donors. We did study the effect of corticosteroids on mRNA and protein expression and could not find a correlation (data not shown). We matched BD subjects with HC subjects on the presence of neurodegenerative disorders (based on amyloid/braak scores), but this was not always possible due to the rarity of the material. Additionally, we excluded donors with CNS autoimmune diseases, such as multiple sclerosis. No information was available about state of the disease ((hypo)mania or depression) during time of death of the donor. This might be an important factor, since monocyte-induced-microglia generated from patients with BD during a manic or depressed stated have been shown to express a different immune profile^62^.

In summary, the present study provides an overview of the immune state of microglia in BD. By using a multi-level approach, microglia were extensively profiled in post-mortem brain tissue of the MFG. Microglia in BD did not display any characteristics indicating immune activation. The data on CX3CR1 expression suggest that other microglial functions important for BD pathogenesis, such as the response to stress and their role in synaptic plasticity, could be affected.

## Supplementary information

Supplementary Material

Supplementary Figure 1

Supplementary Figure 2

Supplementary Figure 3

Supplementary Figure 4
