# Supplementary material for: Microglia in post-mortem brain tissue of patients with bipolar disorder are not immune activated
Source: Transl Psychiatry. 2019 May 24;9:153. doi: 10.1038/s41398-019-0490-x (PMC6534632; doi:10.1038/s41398-019-0490-x)
Supplement: Supplementary file 1 — Supplementary Material [file 41398_2019_490_MOESM1_ESM.docx]

| **Supplementary table 1: clinicopathological information of donors included in the study (1 of 3)** | | | | | | | | | | | | | |
| --- | --- | --- | --- | --- | --- | --- | --- | --- | --- | --- | --- | --- | --- |
| **NBB number** | **Diagnosis** | **Sex** | **Age**  **(years)** | **Braak** | **Amyloid** | **PMD (minutes)** | **pH** | **Medication**  **24h 3 months ever used** | | | **Somatic comorbidity** | **Cause of death** | **Used for** |
| 96/067 | Control | F | 70 | 1 | NA | NA | NA |  |  |  | NA | NA | 1 |
| 96/238 | Control | F | 87 | 2 | O | 480 | 6.91 | NA |  |  | NA | 2 | 2 |
| 96/251 | Control | M | 84 | 1 | NA | 540 | 6.20 |  |  |  | 3 | 2 | 2 |
| 96/373 | Control | M | 70 | 0 | O | 450 | 6.4 | NA | AI |  | 3 | 3 | 1 |
| 97/171 | BD | F | 90 | 3 | B | 390 | NA | AD |  | MS | NA | 2 | 1, 2 |
| 99/258 | BD | M | 68 | 1 | A | 355 | 6.82 | NA |  | AD, MS | 4c | 2 | 1, 2 |
| 00/182 | BD | M | 73 | 2 | B | 315 | 6.38 | NA | AP | AC, AD, MS | 4, 4c | 7 | 2 |
| 00/142 | Control | M | 73 | 0 | O | 1485 | NA | NA | NA | NA | NA | 2 | 1 |
| 00/244 | BD | M | 70 | 1 | O | 290 | 6.26 | NA | NA | AC, AP, MS | 4 | 2, 9 | 1, 2 |
| 01/144 | Control | F | 81 | 3 | C | 425 | 6.30 | NA |  | AP, AD, | 1, 4a, 4c | 1 | 1, 2 |
| 02/031 | BD | M | 68 | 1 | O | NA | 6.64 | NA |  | AP, AD, MS | 4c | 8b | 1, 2 |
| 04/081 | Control | M | 67 | 1 | B | 1115 | 6.70 | NA |  |  | NA | 2 | 1 |
| 05/106 | Control | M | 67 | 5 | C | 250 | 6.4 | B, O | AP, AI |  | 4a | 7 | 1 |
| 06/061 | BD | M | 70 | 3 | C | 383 | 6.53 | B, O | AD, AP, MS |  | 1 | 6 | 1 |
| 06/235 | BD | F | 80 | 1 | B | 570 | 6.33 | AB, AC, AD | AI | MS | 2 | 2 | 1, 2 |
| 08/270 | BD | M | 71 | 1 | O | 385 | 6.49 | NA | AD | MS | 3, 4b | 3 | 1 |
| 09/039 | Control | M | 78 | 1 | O | 1060 | 6.52 | AC, B |  | AD | 4d | 2 | 2 |
| 09/300 | Control | F | 71 | 1 | A | 430 | 6.31 | O |  |  | 4c | 9 | 2 |
| 11/028 | Control | F | 81 | 1 | O | 265 | 6.67 | B, O | AI |  | 2 | 9 | 2 |
| 11/039 | Control | F | 91 | 1 | B | 255 | 6.50 |  |  | NA | 4c | 2 | 2 |
| 11/044 | Control | M | 51 | 0 | O | 465 | 7.05 | NA | NA |  | NA | 5, 7 | 1, 2 |
| 11/069 | Control | M | 49 | 0 | O | 375 | 6.23 | AI | AI | AI | 1,3 | 4 | 2 |
| 11/072 | Control | F | 76 | 2 | O | 435 | 6.87 | B, O | AI |  | 2, 3 | 3, 9 | 2 |
| 11/096 | Control | F | 70 | 2 | A | 375 | 6.55 | AP, B, O | AD, AI |  | 3 | 1, 2, 7 | 1 |
| 12/001 | Control | F | 89 | 2 | B | 340 | 6.75 | B, O |  |  | 1 | 1, 2 | 2 |
| 12/002 | Control | M | 55 | 0 | B | 435 | NA | B, O |  |  | NA | 9 | 2 |
| 12/005 | Control | F | 84 | 2 | A | 336 | 6.68 | AP, B, O | AC |  | 3, 4c | 2 | 1,2 |

| **Supplementary table 1: clinicopathological information of donors included in the study (2 of 3)** | | | | | | | | | | | | | |
| --- | --- | --- | --- | --- | --- | --- | --- | --- | --- | --- | --- | --- | --- |
| **NBB number** | **Diagnosis** | **Gender** | **Age** | **Braak** | **Amyloid** | **PMD (minutes)** | **pH** | **Medication**  **24h 3 months ever used** | | | **Somatic comorbidity** | **Cause of death** | **Used for** |
| 12/048 | BD | M | 81 | NA | NA | 400 | 6.70 |  | AC, AD, AI, MS |  | 1, 3, 4b | 4 | 1, 2 |
| 12/049 | Control | F | 70 | 2 | A | 455 | 6.03 | NA | AC |  | 3 | 3, 7 | 2 |
| 12/059 | Control | F | 78 | 2 | A | 275 | 6.41 | B, O |  |  | 3 | 1 | 2 |
| 12/101 | Control | M | 80 | 2 | C | 265 | 6.59 | O |  |  | 3 | 4 | 1 |
| 12/104 | Control | M | 79 | 2 | A | 390 | 6.71 |  |  |  | NA | 4 | 1 |
| 12/110 | BD | M | 87 | 3 | O | 195 | 6.39 | B, O | AC |  | 4c | 1 | 1, 2 |
| 12/127 | BD | M | 68 | 5 | C | 295 | 6.47 | B, O | AC, AD, AP, MS |  | 4 | 7 | 1, 2 |
| 13/038 | BD | M | 72 | 1 | A | 275 | 6.40 | AP, B, O | AD, MS |  | NA | 8, 9 | 1, 2 |
| 13/056 | Control | M | 95 | 2 | B | 435 | 6.56 | O |  |  | 3 | 2 | 1 |
| 14/005 | Control | M | 67 | NA | NA | 540 | 6.48 | NA | NA |  | NA | NA | 5 |
| 14/020 | Control | F | 93 | 3 | O | 395 | 6.12 | B, O |  |  | NA | 2 | 1 |
| 14/029 | Control | F | 78 | 1 | A | 430 | 6.32 | AD, B, O | NA | NA | 3, 4c | 4 | 1 |
| 14/041 | BD | F | 79 | 2 | B | 480 | 6.31 | B, O | AC, AI, AP, MS |  | 2 | 9 | 1, 2 |
| 14/069 | Control | M | 73 | NA | NA | 265 | 7.00 | NA | NA |  | NA | NA | 5 |
| 14/070 | BD | M | 66 | 1 | B | 455 | 5.83 | AP, B, O | AD, MS | AC | 3 | 3 | 2 |
| 15/027 | Control | F | 76 | 2 | NA | 285 | 6.40 | AP, B, O |  |  | 2, 3 | 3 | 2 |
| 15/031 | BD | F | 51 | 0 | NA | 270 | 6.23 | B, O | AP, MS | MS | 3 | 8b | 1 |
| 15/044 | BD | M | 83 | 1 | A | 925 | 6.50 | NA | AD |  | 4c | 5 | 1, 2 |
| 15/069 | BD | M | 64 | 0 | O | 485 | 6.37 | AB, AP, B | AC | MS | 1, 4c | 1, 7 | 1, 2 |
| 15/075 | BD | M | 58 | NA | NA | 555 | NA | AD | AD | AD | NA | 1, 2 | 1, 3, 5 |
| 15/077 | BD | M | 72 | 1 | A | 595 | 6.47 | NA | AC, AD | AI, MS | NA | 1, 2 | 1, 2, 3 |
| 15/087 | Control | F | 75 | NA | NA | 550 | 6.57 |  |  |  | NA | 4 | 3, 4, 5 |
| 15/089 | Control | F | 92 | NA | NA | 465 | 6.71 |  |  |  | NA | 4 | 4, 5 |
| 15/101 | BD | F | 92 | NA | NA | NA | NA |  |  | AP, AD | 4a, 4d | 2 | 3, 5 |
|  |  |  |  |  |  |  |  |  |  |  |  |  |  |

| **Supplementary table 1: clinicopathological information of donors included in the study (3 of 3)** | | | | | | | | | | | | | |
| --- | --- | --- | --- | --- | --- | --- | --- | --- | --- | --- | --- | --- | --- |
| **NBB number** | **Diagnosis** | **Gender** | **Age** | **Braak** | **Amyloid** | **PMD (minutes)** | **pH** | **Medication**  **24h 3 months ever used** | | | **Somatic comorbidity** | **Cause of death** | **Used for** |
| 15/107 | BD | F | 77 | NA | NA | NA | NA | AP, B, O |  | AD, MS | 2 | 9 | 3, 4, 5 |
| 16/024 | BD | F | 88 | NA | NA | 600 | 6.26 | AP, B, O | AD |  | 3 | 7 | 3, 4, 5 |
| 16/027 | Control | M | 70 | NA | NA | 525 | 6.35 | NA | NA | NA | NA | 3 | 3, 4, 5 |
| 16/033 | BD | M | 85 | NA | NA | 525 | 6.28 | AD, MS |  |  |  | 4 | 3, 4 |
| 16/038 | Control | F | 85 | NA | NA | 425 | 6.52 | NA | NA | NA |  | 1 | 3, 4, 5 |
| 16/046 | Control | F | 92 | NA | NA | 411 | 6.60 |  |  |  |  | 9 | 3, 4, 5 |
| 16/056 | Control | M | 68 | NA | NA | 350 | 6.50 | AP, B, O |  |  |  | 3 | 3, 4, 5 |
| 16/065 | BD | F | 93 | NA | NA | 310 | 7.55 | AP |  | MS | 2, 3 | 2 | 3, 4, 5 |
| 16/067 | Control | M | 89 | NA | NA | 492 | 6.59 | NA | NA | NA |  | 2 | 4 |
| 16/078 | Control | F | 84 | NA | NA | 460 | 7.50 | NA | NA | NA |  | 9 | 3, 4, 5 |
| 16/080 | Control | M | 83 | NA | NA | 305 | 7.12 |  |  | AI | 3 | 4 | 3, 4, 5 |
| 16/116 | Control | F | 81 | NA | NA | 315 | NA |  |  |  |  | 8a | 3, 4, 5 |
| 16/137 | Control | M | 77 | NA | NA | 765 | 6.46 |  |  |  | 3 | 2 | 3, 4, 5 |
| 17/003 | Control | F | 96 | NA | NA | 315 | 6.71 | O |  |  | 4a | 7 | 3, 4, 5 |
| 17/004 | BD | F | 45 | NA | NA | 450 | 6.93 |  |  | AP |  | NA | 3, 4, 5 |
| 17/005 | Control | F | 60 | NA | NA | 330 | 7.07 |  | AD | AI |  | 4 | 3, 4, 5 |
| 17/009 | BD | F | 46 | NA | NA | 345 | NA | AB, AC, MS |  | AD |  | 4 | 3, 4, 5 |
| 17/043 | Control | M | 80 | NA | NA | 570 | NA |  |  |  | 4a | 9 | 3 |
| 17/078 | Control | F | 88 | NA | NA | 600 | 6.66 |  |  |  | 2, 4b, 4d | 2 | 3, 4, 5 |
| 17/097 | Control | F | 83 | NA | NA | 410 | 6.75 |  | AI | AI | 2 | 4 | 3, 4, 5 |
| 17/102 | Control | F | 98 | NA | NA | 365 | 6.92 |  | AP |  |  | 4 | 3, 4, 5 |
| 17/124 | Control | F | 55 | NA | NA | 450 | NA |  | AC, AD |  |  | 4 | 3, 5 |
| 17/128 | BD | M | 90 | NA | NA | 380 | NA |  |  | AI, MS | 4a, 4d | 9 | 3 |
| 17/148 | BD | M | 71 | NA | NA | 340 | 6.54 |  |  | AC, AP, MS | 4a | 4 | 3, 4, 5 |
| 18/018 | Control | F | 82 | NA | NA | 330 | 6.48 | B,O |  |  |  | 1 | 5 |
| 18/021 | Control | M | 92 | NA | NA | 530 | 6.89 | O |  | AD |  | 4 | 4 |
| 18/039 | BD | F | 82 | NA | NA | 615 | 6.60 | AC, AP |  | AD, MS |  | 2 | 3, 4, 5 |
| **NBB =** Netherlands brain bank. **Control** = control donor; **BD** = donor with bipolar disorder; **M** = male; **F** = female; **NA** = not applicable; **PMD** = post-mortem delay; **medication**: If medication was used 24 hours prior to death, it has not been reported again at 3 months or ever used. If medication is used 3 months prior to death, it has not been reported again at ever used. AB = antibiotics (only reported at 24h); AC = anticonvulsants; AD = antidepressants; AI = anti-inflammatory (corticosteroids); AP = antipsychotics; B = benzodiazepines (only reported at 24h); MS = mood stabilizers; O = opiates (only reported at 24h); **somatic** **comorbidity**: 1 = infection < 2 weeks prior to death; 2 = auto-immune disease; 3 = cancer (in history); 4 = neuropathology; a = Dementia; b = Parkinson; c = ischemia/infarction; d = bleeding (CVA, TIA); **cause of death**: 1 = infection/inflammatory; 2 = cardiorespiratory; 3 = cancer; 4 = euthanasia/palliative sedation*; 5 = suicide; 6 = trauma; 7 = cachexia/dehydration; 8 = brain; a = ischemia/infarction; b = bleeding (CVA, TIA); 9 = other (ileus, organ failure); **used for**: 1 = immunochemistry (paraffin tissue); 2 = mRNA expression (frozen tissue); 3 = mRNA expression (isolated microglia); 4 = protein expression (isolated microglia); 5 = LPS response (isolated microglia); * euthanasia is legal according to Dutch law. | | | | | | | | | | | | | |

| **Supplementary table 2: primer sequence for qPCR analysis** | | | | | |
| --- | --- | --- | --- | --- | --- |
| **Primer** | | **Forward sequence** | | **Reverse sequence** | |
| *18S* | | TAGTCGCCGTGCCTACCAT | | CCTGCTGCCTTCCTTGGA | |
| *ACTB* | GTGGACATCCGCAAAGACCT | | TCTGCATCCTGTCGGCAAT | |  |
| *AIF1* | | AGACGTTCAGCTACCCTGACTT | | GGCCTGTTGGCTTTTCCTTTTCTC | |
| *CD68* | | CTTCTCTCATTCCCCTATGGACA | | GAAGGACACATTGTACTCCACC | |
| *CD163* | | TTTGTCAACTTGAGTCCCTTCAC | | TCCCGCTACACTTGTTTTCAC | |
| *CX3CR1* | | CTTACGATGGCACCCAGTGA | | CAAGGCAGTCCAGGAGAGTT | |
| *GAPDH* | | TGCACCACCAACTGCTTAGC | | GGCATGGACTGTGGTCATGA | |
| *HLA-DRA* | | CCCAGGGAAGACCACCTTT | | CACCCTGCAGTCGTAAACGT | |
| *IL1B* | | TTTGAGTCTGCCCAGTTCCC | | TCAGTTATATCCTGGCCGCC | |
| *IL6* | | TGCAATAACCACCCCTGACC | | TGCGCAGAATGAGATGAGTTG | |
| *ITGAM* | | TGCTTCCTGTTTGGATCCAACCTA | | AGAAGGCAATGTCACTATCCTCTTGA | |
| *MRC1* | | TGCAGAAGCAAACCAAACCTGTAA | | CAGGCCTTAAGCCAACGAAACT | |
| *P2RY12* | | TTTGTGTGTCAAGTTACCTCCG | | CTGGTGGTCTTCTGGTAGCG | |
| *SDHA* | | GAAGCCCTTTGAGGAGCACT | | GTTTTGTCGATCACGGGTCT | |
| *TMEM119* | | CTTCCTGGATGGGATAGTGGAC | | GCACAGACGATGAACATCAGC | |

| **Supplementary table 3: flow cytometry antibodies and isotype controls** | | | | |
| --- | --- | --- | --- | --- |
| **Antibody** | **Label** | **Clone** | **Species** | **Manufacturer** |
| CD11c | FITC | 3.9 | mouse | Thermo Fisher Scientific |
| CD16 | FITC | LNK16 | mouse | BIO RAD |
| CD32 | FITC | 6C4 | mouse | Thermo Fisher Scientific |
| CD45 | FITC | 2D1 | mouse | Thermo Fisher Scientific |
| CD200R | FITC | OX108 | mouse | BIO RAD |
| CD11b | PE | ICRF44 | mouse | Thermo Fisher Scientific |
| CD40 | PE | 5C3 | mouse | Thermo Fisher Scientific |
| CD83 | PE | HB15e | mouse | Thermo Fisher Scientific |
| CD86 | PE | IT2.2 | mouse | Thermo Fisher Scientific |
| CD163 | PE | eBioGHI/61 | mouse | Thermo Fisher Scientific |
| CD14 | APC | 61D3 | mouse | Thermo Fisher Scientific |
| CD64 | APC | 10.1 | mouse | Thermo Fisher Scientific |
| CD172α | APC | 15-414 | mouse | Thermo Fisher Scientific |
| CD206 | APC | 19.2 | mouse | BD biosciences |
| CX3CR1 | APC | 2A9-1 | rat | Thermo Fisher Scientific |
| HLA-DR | APC | LN3 | mouse | Thermo Fisher Scientific |
| IgG1 | FITC |  | mouse | Thermo Fisher Scientific |
| IgG1 | APC |  | mouse | Thermo Fisher Scientific |
| IgG2a | APC |  | mouse | Thermo Fisher Scientific |
| IgG2b | APC |  | rat | Thermo Fisher Scientific |
| IgG2b | PE |  | mouse | Biolegend |
| IgG1 | PE |  | mouse | Biolegend |
| IgG2b | APC |  | mouse | Biolegend |
| Thermo Fisher Scientific, Massachusetts, USA; BIO RAD, California, USA; BD biosciences, New Jersey, USA; Biolegend, California, USA. | | | | |

|  |  |  | **Gender** | | **Age** | | **PMD** | | **pH** | |
| --- | --- | --- | --- | --- | --- | --- | --- | --- | --- | --- |
|  |  |  | **Spearman rho** | **N** | **Spearman rho** | **N** | **Spearman rho** | **N** | **Spearman rho** | **N** |
| **Immunohistochemistry**  (paraffin tissue) | medial frontal gyrus | **microglial density** | **-.317*** | 53 | 0.170 | 53 | 0.021 | 51 | -0.189 | 49 |
|  |  | **microglial morphology** | 0.248 | 49 | 0.301 | 49 | 0.222 | 47 | 0.170 | 45 |
| **mRNA expression** (frozen tissue) | medial frontal gyrus | ***AIF1*** | -0.262 | 57 | 0.114 | 57 | -0.222 | 55 | 0.258 | 54 |
|  |  | ***P2Y12*** | 0.084 | 57 | 0.070 | 57 | -0.065 | 55 | 0.292 | 54 |
|  |  | ***TMEM119*** | -0.155 | 57 | 0.099 | 57 | -0.281 | 55 | 0.293 | 54 |
|  |  | ***CD68*** | -0.260 | 57 | 0.232 | 57 | -0.217 | 55 | 0.326 | 54 |
|  |  | ***ITGAM*** | -0.213 | 57 | 0.177 | 57 | -0.336 | 55 | 0.288 | 54 |
|  |  | ***IL1B*** | -0.299 | 57 | 0.181 | 57 | -0.251 | 55 | **.376*** | 54 |
|  |  | ***IL6*** | -0.348 | 57 | 0.238 | 57 | -0.092 | 55 | 0.044 | 54 |
|  |  | ***HLA-DRA*** | -0.301 | 57 | 0.153 | 57 | -0.100 | 55 | 0.363 | 54 |
|  |  | ***CX3CR1*** | -0.020 | 60 | 0.057 | 60 | -0.132 | 58 | **.467**** | 57 |
| **mRNA expression** (isolated microglia) | medial frontal gyrus | ***IL1B*** | 0.027 | 23 | 0.146 | 23 | **.435*** | 21 | -0.143 | 16 |
|  |  | ***IL6*** | -0.013 | 23 | 0.318 | 23 | 0.326 | 21 | -0,290 | 16 |
|  |  | ***CD163*** | 0.054 | 23 | 0.228 | 23 | 0.408 | 21 | -0.346 | 16 |
|  |  | ***MRC1*** | -0.013 | 23 | 0.318 | 23 | 0.326 | 21 | 0.035 | 16 |
|  |  | ***TMEM119*** | -0.215 | 23 | -0.242 | 23 | -0.126 | 21 | 0.049 | 16 |
|  |  | ***CX3CR1*** | -0.201 | 23 | -0.135 | 23 | -0.105 | 21 | **-.528*** | 16 |
|  | superior temporal gyrus | ***IL1B*** | 0.175 | 23 | -0.055 | 23 | **.461*** | 23 | -0.024 | 18 |
|  |  | ***IL6*** | -0.389 | 23 | -0.146 | 23 | 0.210 | 23 | 0.198 | 18 |
|  |  | ***CD163*** | 0.376 | 23 | 0.134 | 23 | **.468*** | 23 | **-.611**** | 18 |
|  |  | ***MRC1*** | 0.054 | 23 | -0.288 | 23 | 0.230 | 23 | -0.105 | 18 |
|  |  | ***TMEM119*** | -0.134 | 23 | 0.031 | 23 | -0.375 | 23 | 0.032 | 18 |
|  |  | ***CX3CR1*** | -0.317 | 23 | -0.270 | 23 | -0.003 | 23 | 0.384 | 18 |
|  | thalamus | ***IL1B*** | -0.400 | 21 | 0.006 | 21 | 0.224 | 20 | -0.202. | 16 |
|  |  | ***IL6*** | -0.009 | 20 | -0.327 | 20 | 0.365 | 19 | -0.057 | 15 |
|  |  | ***CD163*** | 0.334 | 21 | 0.407 | 21 | 0.165 | 20 | **-0.693**** | 16 |
|  |  | ***MRC1*** | -0.100 | 20 | -0.249 | 20 | 0.336 | 19 | -0.141 | 15 |
|  |  | ***TMEM119*** | -0.064 | 20 | -0.132 | 20 | -0.207 | 19 | -0.007 | 15 |
|  |  | ***CX3CR1*** | -0.300 | 20 | **-.463*** | 20 | -0.004 | 19 | **0.531*** | 15 |

**Supplementary table 4: Spearman rho correlations of confounding variables gender, age, PMD and pH (1 of 2)**

**Supplementary table 4: Spearman rho correlations of confounding variables gender, age, PMD and pH (2 of 2)**

|  |  |  | **Gender** | | **Age** | | **PMD** | | **pH** | |
| --- | --- | --- | --- | --- | --- | --- | --- | --- | --- | --- |
|  |  |  | **Spearman rho** | **N** | **Spearman rho** | **N** | **Spearman rho** | **N** | **Spearman rho** | **N** |
| **protein expression** (isolated microglia) | medial frontal gyrus | **CD11b** | 0.294 | 24 | 0.163 | 24 | -0.158 | 23 | 0.252 | 21 |
|  |  | **CD45** | 0.306 | 24 | -0.101 | 24 | 0.307 | 23 | -0.322 | 21 |
|  |  | **HLA-DR** | 0.281 | 24 | 0.203 | 24 | 0.084 | 23 | -0.040 | 21 |
|  |  | **CX3CR1** | 0.217 | 24 | 0.044 | 24 | 0.187 | 23 | -0.-028 | 21 |
|  | superior temporal gyrus | **CD11b** | -0.022 | 26 | 0.001 | 26 | -0.394 | 26 | 0.329 | 23 |
|  |  | **CD45** | 0.340 | 26 | 0.130 | 26 | 0.321 | 25 | -0.405 | 23 |
|  |  | **HLA-DR** | 0.156 | 26 | 0.207 | 26 | -0.152 | 25 | 0.133 | 23 |
|  |  | **CX3CR1** | 0.307 | 26 | 0.190 | 26 | 0.037 | 25 | 0.090 | 23 |
|  | thalamus | **CD11b** | 0.226 | 25 | 0.005 | 25 | -0.314 | 24 | 0.309 | 22 |
|  |  | **CD45** | 0.167 | 25 | 0.302 | 25 | -0.137 | 24 | 0.115 | 22 |
|  |  | **HLA-DR** | -0.119 | 25 | 0.008 | 25 | -0.055 | 24 | 0.244 | 22 |
|  |  | **CX3CR1** | 0.131 | 25 | -0.025 | 25 | 0.017 | 24 | 0.275 | 22 |
| **LPS response** (isolated microglia) | medial frontal gyrus | ***IL1B*** | -0.025 | 25 | -0.089 | 25 | **-.743**** | 23 | 0.348 | 19 |
|  |  | ***IL6*** | 0.099 | 25 | -0.212 | 25 | -.415* | 23 | 0.428 | 19 |
|  |  | ***TNF*** | 0.037 | 25 | -0.099 | 25 | **-.542*** | 23 | **.642**** | 19 |
|  | superior temporal gyrus | ***IL1B*** | 0.194 | 12 | 0.007 | 12 | -0.491 | 11 | 0.267 | 10 |
|  |  | ***IL6*** | 0.000 | 11 | -0.347 | 11 | -0.370 | 11 | 0.527 | 10 |
|  |  | ***TNF*** | 0.259 | 12 | -0.2549 | 12 | -0.136 | 11 | -0.067 | 10 |
|  | thalamus | ***IL1B*** | -0.315 | 17 | 0.3434 | 17 | -0.363 | 15 | 0.512 | 13 |
|  |  | ***IL6*** | -0.031 | 17 | 0.100 | 17 | -0.483 | 15 | **.635*** | 13 |
|  |  | ***TNF*** | 0.063 | 17 | 0.108 | 17 | -0.370 | 15 | 0.470 | 13 |
| Spearman rho correlations, *p*-values and number of donors included (N) of the four confounding variables (gender, age, post-mortem delay (PMD) and pH) on different experiments in the study. Significant values after Bonferroni correction for multiple testing are highlighted in red. In case of significant associations, ANCOVA was applied for further analysis to correct the confounding effect.  * *p*<0.05; ** *p*<0.01 | | | | | | | | | | |

| **Supplementary table 5: geomean fluorescent intensity (geoMFI) of isolated microglia** | | | | | | | | | |
| --- | --- | --- | --- | --- | --- | --- | --- | --- | --- |
|  | **Medial frontal gyrus (MFG)** | | | **Superior temporal gyrus (STG)** | | | **Thalamus (THA)** | | |
| **Marker** | **Control (N=17)** | **BD (N=7)** | ***p*-value** | **Control (N=17)** | **BD (N=9)** | ***p*-value** | **Control (N=16)** | **BD (N=9)** | ***p*-value** |
| **CD11b** | 669.5 ± 131.0 | 1219.7 ± 705.7 | 0.90 | 604.6 ± 119.5 | 544.1 ± 172.1 | 0.88 | 771.6 ± 156.9 | 910.8 ± 297.4 | 0.89 |
| **CD11c** | 356.8 ± 44.1 | 235.8 ± 96.3 | 0.09 | 338.8 ± 58.0 | 453.6 ± 164.7 | 0.99 | 346.4 ± 50.9 | 389.3 ± 97.8 | 0.90 |
| **CD14** | 241.9 ± 66.4 | 419.6 ± 267.2 | 0.89 | 211.2 ± 66.3 | 156.0 ± 58.7 | 0.61 | 227.4 ± 67.4 | 373.0 ± 131.1 | 0.38 |
| **CD16** | 110.0 ± 31.5 | 39.8 ± 32.7 | 0.09 | 159.9 ± 43.7 | 41.4 ± 35.8 | 0.05 | 135.1 ± 42.5 | 89.1 ± 47.9 | 0.49 |
| **CD32** | 557.7 ± 143.8 | 1839.6 ± 992.9 | 0.75 | 513.9 ± 147.0 | 1276.0 ± 1080.0 | 0.37 | 599.8 ± 202.7 | 1555.0 ± 859.8 | 0.85 |
| **CD40** | 13.1 ± 5.3 | 1.4 ± 1.4 | 0.20 | 9.3 ± 3.7 | 5.3 ± 3.6 | 0.44 | 11.7 ± 5.3 | 8.6 ± 7.3 | 0.51 |
| **CD45** | 714.8 ± 101.5 | 1132.1 ± 321.9 | 0.35 | 813.7 ± 125.8 | 1081.0 ± 244.2 | 0.49 | 789.1 ± 80.3 | 978.8 ± 215.4 | 0.85 |
| **CD64** | 2152.3 ± 469.9 | 2292.3 ± 961.3 | 0.95 | 1975.0 ± 355.7 | 1351.0 ± 442.5 | 0.21 | 2011.0 ± 357.8 | 1716.0 ± 701.2 | 0.31 |
| **CD83** | 42.4 ± 31.9 | 0.7 ± 0.7 | 0.35 | 56.9 ± 44.5 | 6.2 ± 6.0 | 0.21 | 75.2 ± 63.3 | 4.0 ± 2.1 | 0.92 |
| **CD86** | 76.8 ± 18.5 | 141.4 ± 58.0 | 0.39 | 96.4 ± 24.3 | 115.7 ± 49.5 | 0.86 | 109.9 ± 30.2 | 133.4 ± 45.6 | 0.77 |
| **CD163** | 49.0 ± 48.1 | 15.7 ± 13.1 | 0.48 | 0.7 ± 0.6 | 2.2 ± 2.2 | >0.99 | 7.8 ± 4.1 | 1.7 ± 1.2 | 0.45 |
| **CD172α** | 586.9 ± 88.9 | 376.2 ± 120.5 | 0.21 | 556.1 ± 104.4 | 310.8 ± 117.3 | 0.19 | 738.1 ± 153.2 | 636.8 ± 171.0 | 0.85 |
| **CD200R** | 422.1 ± 156.4 | 361.4 ± 176.0 | 0.61 | 495.2 ± 209.8 | 1034.0 ± 520.7 | 0.37 | 404.9 ± 156.3 | 473.8 ± 187.7 | 0.72 |
| **CD206** | 64.3 ± 53.0 | 5.1 ± 3.1 | 0.96 | 11.4 ± 6.5 | 5.3 ± 3.1 | 0.94 | 25.4 ± 12.8 | 20.7 ± 17.9 | 0.99 |
| **CX3CR1** | 531.0 ± 78.4 | 694.3 ± 162.3 | 0.21 | 504.5 ± 74.9 | 249.1 ± 75.8 | 0.04 | 474.1 ± 82.6 | 452.3 ± 118.5 | 0.90 |
| **HLA-DR** | 1374.2 ± 281.1 | 1889.7 ± 658.6 | 0.66 | 1279.0 ± 308.2 | 915.4 ± 241.1 | 0.71 | 1418.0 ± 275.3 | 1532.0 ± 368.7 | 0.76 |
| Protein expression of isolated microglia from the medial frontal gyrus, superior temporal gyrus and thalamus from controls and patients with bipolar disorder (BD), determined by flow cytometry. Mean fluorescent intensity is visualized as mean ± standard error of the mean. Non-parametric testing was used to test differences between controls and patients with BD. Red *p*=values are significant before Bonferroni correction. | | | | | | | | | |

**Supplementary table 6 Differential expression of several microglia-specific genes in bipolar disorder**

|  | **Gandal *et al.* RNAseq** | | | **Gandal *et al.*microglia array** | | |
| --- | --- | --- | --- | --- | --- | --- |
|  | log2FC | P-value | FDR | log2FC | P-value | FDR |
| *AIF1* | -0,171 | **0,039*** | 0,238 | -0,184 | **0,000*** | **0,019**** |
| *CX3CR1* | -0,400 | **6,99E-05*** | **0,007**** | -0,271 | **0,033*** | 0,200 |
| *IL1B* | 0,238 | 0,084 | 0,350 | 0,026 | 0,621 | 0,820 |
| *IL6* | 0,075 | 0,659 | 0,874 | 0,030 | 0,652 | 0,836 |
| *P2RY12* | -0,337 | **4,67E-05*** | **0,006**** | NA | NA | NA |
| *HLA-DRA* | -0,130 | 0,117 | 0,411 | -0,209 | **0,020*** | 0,155 |
| *ITGAM* | -0,140 | **0,027*** | 0,199 | -0,155 | **0,000*** | **0,017**** |
| *CD68* | -0,091 | 0,288 | 0,626 | NA | NA | NA |
| *MRC1* | -0,367 | 0,089 | 0,362 | NA | NA | NA |
| *CD163* | 0,036 | 0,744 | 0,913 | 0,013 | 0,810 | 0,916 |
| *TMEM119* | -0,228 | **0,002*** | 0,054 | NA | NA | NA |

Abbrevations. FDR=false discovery rate. NA=not applicable.

**Supplementary Methods**

*Quantification of cell number*

The number of microglia per picture was quantified using a macro in ImageJ software on sections of the medial frontal gyrus of patients with bipolar disorder and controls. The macro consisted of the following steps: transformation to 8-bit; scale scaling pixels to μm (according to microscope guidelines); automated default threshold applied; minimum radius mask of 1 and maximum radius mask of 3; converted to mask; particles filtered with size > 45; option “outline” and “exclude cells at edges”.

*Macro for the number of cell bodies per picture***:**

run("8-bit");

run("Set Scale...", "distance=… known=1 pixel=1 unit= μm global");

setAutoThreshold("Moments");

//run("Threshold...");

setAutoThreshold("Moments");

getThreshold(lower, upper);

setThreshold(lower,upper-35);

setOption("BlackBackground", false);

run("Minimum...", "radius=1");

run("Maximum...", "radius=3");

run("Convert to Mask");

run("Analyze Particles...", "size=45-Infinity circularity=0.00-1.00 pixel show=Outlines exclude clear include summarize");

selectWindow("Drawing of " + name);

saveAs("Tiff", output + name + " Cell Body Mask");

}

*Quantification of microglial cell density*

Per tissue section, six pictures (89.44 µm x 119.37 µm) were taken randomly from grey matter and white matter separately and blinded for diagnosis. To determine the microglial cell density, the cell body mask was used to count the total number of microglial cells in each taken picture. Thereafter, the average number of microglial cells of six pictures was calculated and divided by the area of the pictures.

*Quantification of microglia/total cell count*

The total number of Hoechst+ cells per immunofluorescent picture was quantified using a particle analysis macro script in Image J: automated default threshold applied; particles were quantified with size of > 0.01 pixels ; positive area was summarized. To determine the fraction of IBA-1+Hoechst+ cells the following RG2B co-localization macro plugin was used in ImageJ: automated default threshold applied; minimum threshold = 0, co-localized pixels maximum threshold was set to 80 pixels for red channel and 60 pixels for green channel, particles were quantified with size of > 150 pixels; positive area was summarized; co-localization data are displayed as a RGB image. The microglial density was defined as the number of microglia/total cell count ratio, by dividing the average number of microglia (IBA1+Hoechst+ cells) by the average total number of Hoechst+ cells.

*Macro for the particle analysis:*

{

run("Split Channels");

run("Duplicate...", " ");

setAutoThreshold("Moments dark");

run("Convert to Mask");

run("Analyze Particles...", "size=0.01-Infinity show=[Bare Outlines] display exclude clear include summarize add");

selectWindow(name + " (blue)-1");

close();

selectWindow("Drawing of "+ name + " (blue)-1");

close();

selectWindow(name + " (blue)");

close();

selectWindow("Results");

close();

}

*Macro for the co-localization* {

//run("Channels Tool...");

run("Make Composite");

Stack.setDisplayMode("color");

Stack.setChannel(3);

run("Red");

Stack.setDisplayMode("composite");

run("Stack to RGB");

run("RG2B Colocalization", " minimum=0 red=80 green=60 set=[the max of the red and green] display=[as an RGB image]");

run("8-bit");

//run("Threshold...");

setAutoThreshold("Moments dark");

call("ij.plugin.frame.ThresholdAdjuster.setMode", "Red");

//setThreshold(60, 255);

setOption("BlackBackground", false);

run("Convert to Mask");

run("Analyze Particles...", "size=150-Infinity show=[Bare Outlines] display exclude clear include summarize add");

selectWindow(name + " (RGB)");

saveAs("Tiff", output + name + " Colocalization Original File");

selectWindow("RG2B Colocalization - " + name + " (RGB)");

saveAs("Tiff", output + name + " Colocalization RG2B ");

selectWindow("Results");

saveAs("Results", output + name + " Colocalization Results.txt");

close();

selectWindow("Colocalization Data - " + name + " (RGB)");

saveAs("Tiff", output + name + " Colocalization Mask");

close();

selectWindow("Drawing of Colocalization Data - " + name + " (RGB)");

saveAs("Tiff", output + name + " Colocalization Mask Drawing");

close();

}

**Supplementary figures**

**Supplementary figure 1: Microglial density and morphological analysis.** Microglial density and morphological analysis in the medial frontal gyrus of patients with bipolar disorder (BD) and controls in both grey and white matter. **a)** Immunofluorescent staining was performed additionally to determine the number of microglia relative to total cell number (I). Staining procedure was highly similar as for DAB-immunostained microglia, with the usage of donkey anti-rabbit Alexa 488 (1:700) and Hoechst (1:1000) as secondary antibody. An automated macro script was used to quantify the number of microglia (Iba1^+^Hoechst^+^; II) or total cell number (Hoechst^+^; III). Square shows zoom-in of a single cell. **b)** Quantification of the number of microglia relative to total cell number in grey and white matter of patients with BD (red circles) and controls (blue dots), displayed as the ratio of microglia divided by the total cell number. **c)** Visual representation of the automated analysis with ImageJ to determine microglial density. The threshold was adjusted to generate a cell body mask (IV, V) representing the microglia cell density.

**Supplementary figure 2: mRNA expression profile of isolated microglia from superior temporal gyrus and thalamus**. mRNA expression was determined in microglia isolated microglia from the superior temporal gyrus **(a-f)** and thalamus **(g-l)** in patients with bipolar disorder (BD, red circles) and controls (blue dots). mRNA expression of *IL1B* **(a, g)**, *IL6* **(b, h)**, *CD163* **(c, i)**, *MRC1* **(d, j)**, *TMEM119* **(e, k)**, and *CX3CR1* (**f, l**) by qPCR. Distinction was made between pro-inflammatory genes *IL1B* **(a, g)** and *IL6* **(b, h)**, anti-inflammatory genes *CD163* **(c, i)** and *MRC1* **(d, j)**, and the microglial specific genes *TMEM119* **(e, k)**, and *CX3CR1* (**f, l**) mRNA expression was normalized to β-Actin (*ACTB*) and glyceraldehyde 3-phosphate dehydrogenase (GAPDH) using the ΔΔCT method. ND = number of non-detected samples

**Supplementary figure 3: Protein expression profile of isolated microglia from superior temporal gyrus and thalamus**. Protein expression, determined by flow cytometry, of microglia isolated from the superior temporal gyrus **(a-d)** and thalamus **(e-h)** from controls (N=17, blue dots) and patients with bipolar disorder (BD, N=9, red circles). Geomean fluorescent intensity (MFI) is shown for CD11b **(a, e)**, CD45 **(b, f)**, HLA-DR **(c, g)**, and CX3CR1 **(d, h)**.

**Supplementary figure 4: LPS response of isolated microglia from superior temporal gyrus and thalamus.** LPS response of human primary microglia isolated from the superior temporal gyrus (STG; a) and thalamus (THA; b) in controls (blue dots) and patients with bipolar disorder (BD, red circles). mRNA expression of *IL1B*, *IL6,* and *TNF* expression is determined with qPCR. The fold change was calculated by dividing mRNA expression of the LPS stimulated sample by mRNA expression of the non-stimulated sample of the same subject. The dotted line represents baseline mRNA expression of non-stimulated cells.
