# Supplementary figures and images for: Microglia in post-mortem brain tissue of patients with bipolar disorder are not immune activated
Source: Transl Psychiatry. 2019 May 24;9:153. doi: 10.1038/s41398-019-0490-x (PMC6534632; doi:10.1038/s41398-019-0490-x)

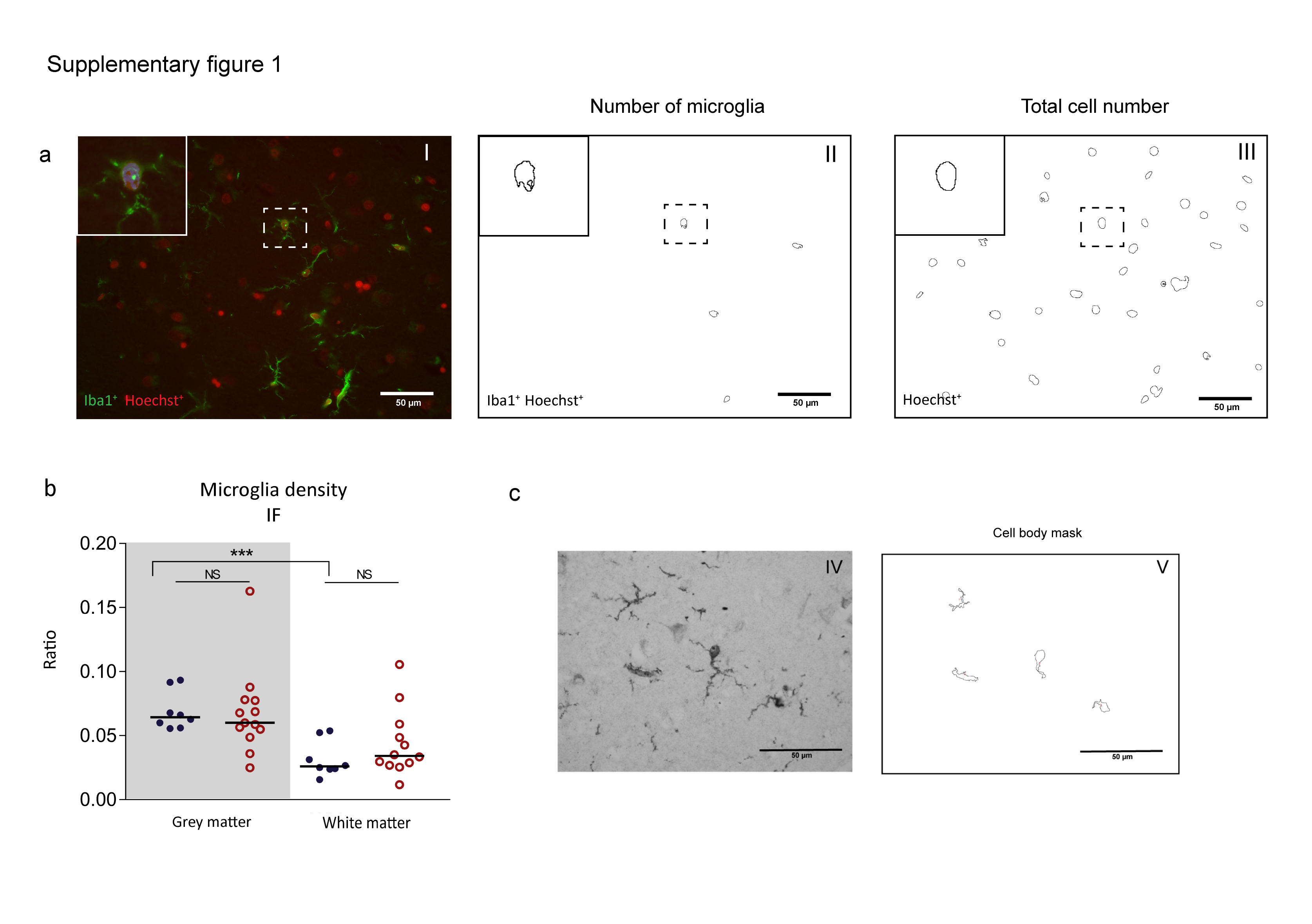

Supplement: Supplementary file 2 — Supplementary Figure 1 [file 41398_2019_490_MOESM2_ESM.png]

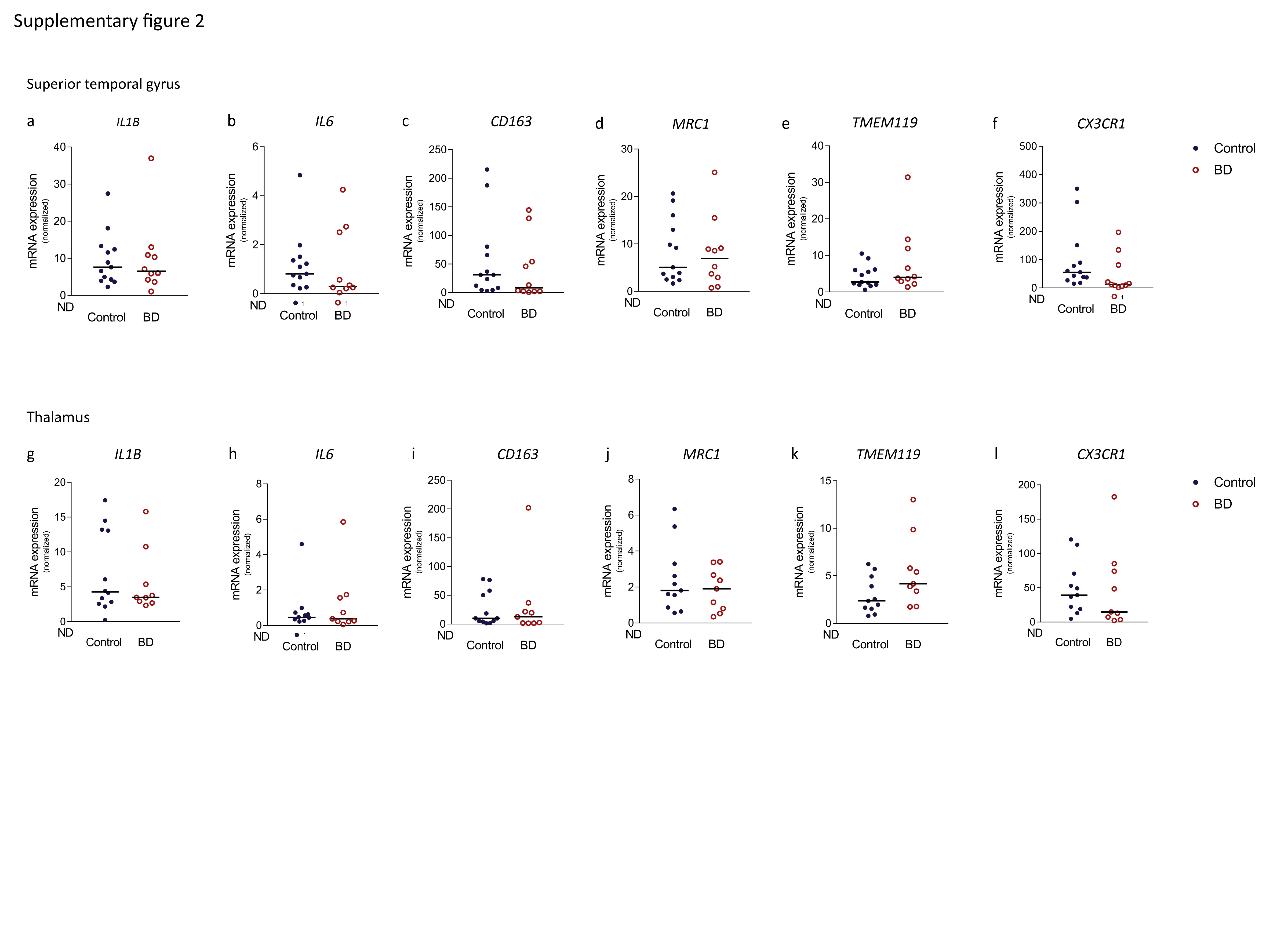

Supplement: Supplementary file 3 — Supplementary Figure 2 [file 41398_2019_490_MOESM3_ESM.png]

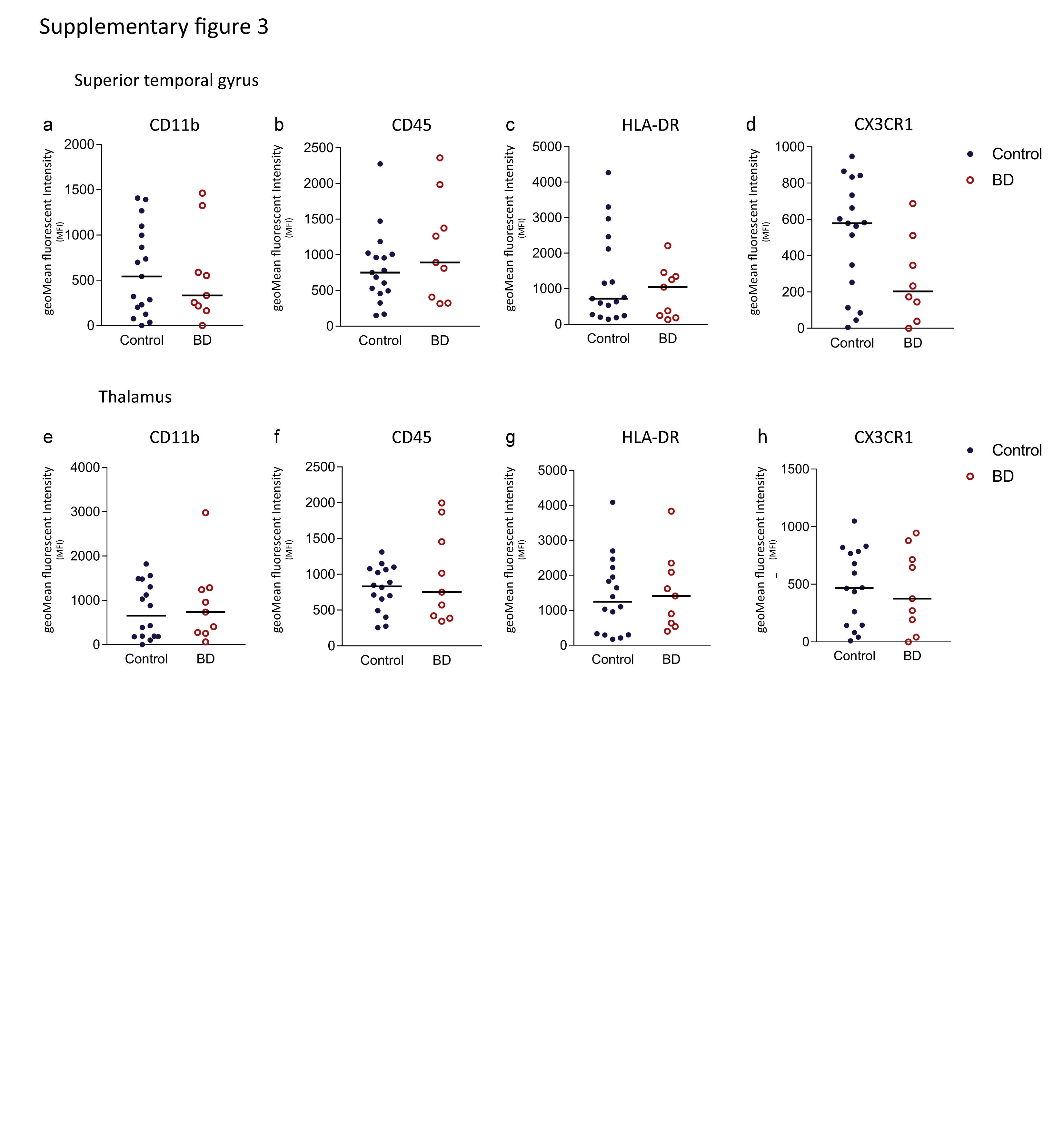

Supplement: Supplementary file 4 — Supplementary Figure 3 [file 41398_2019_490_MOESM4_ESM.png]

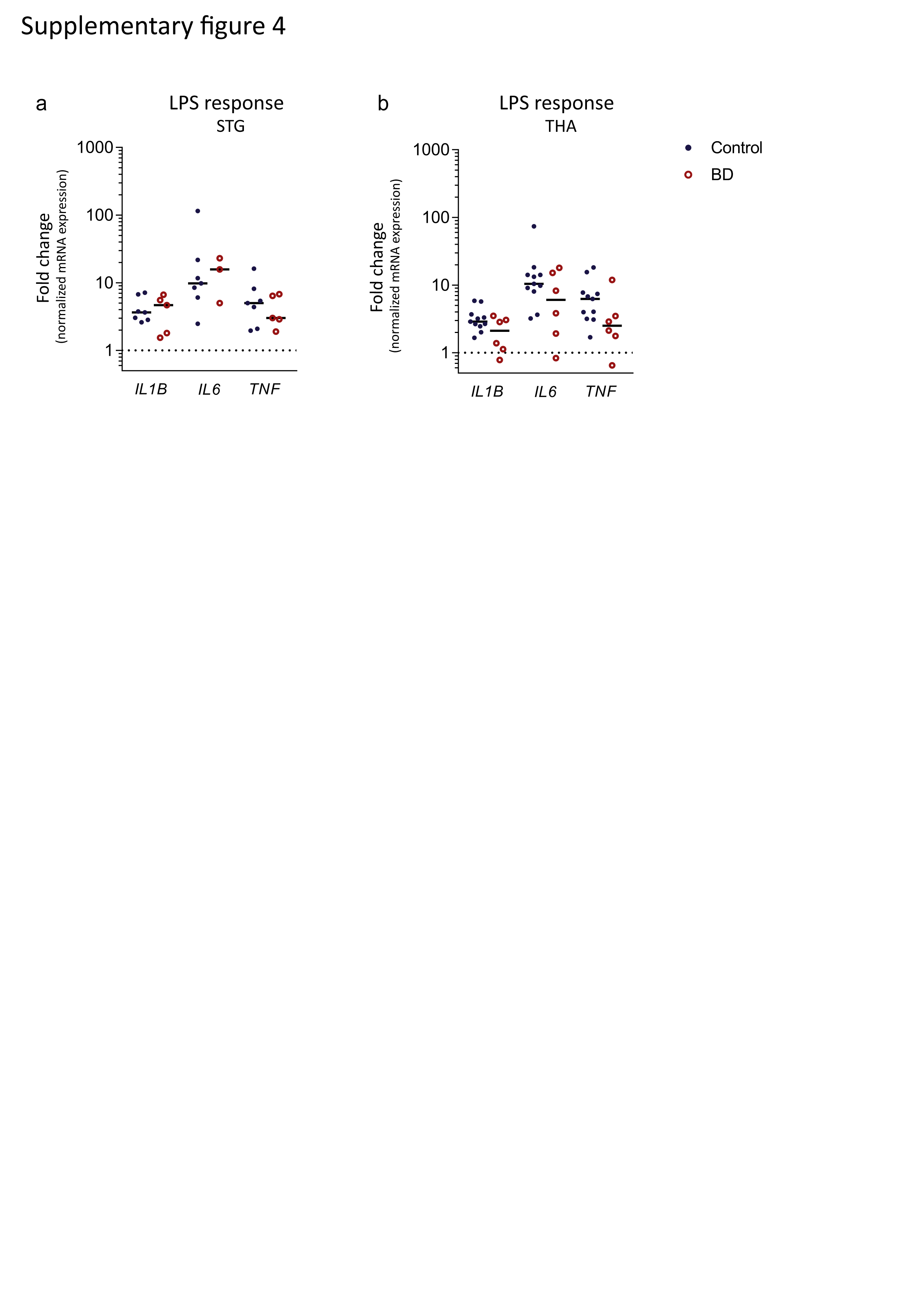

Supplement: Supplementary file 5 — Supplementary Figure 4 [file 41398_2019_490_MOESM5_ESM.png]
